# The Past, Present and Future of *Cannabis sativa* Tissue Culture

**DOI:** 10.3390/plants10010185

**Published:** 2021-01-19

**Authors:** Adrian S. Monthony, Serena R. Page, Mohsen Hesami, Andrew Maxwell P. Jones

**Affiliations:** Department of Plant Agriculture, Gosling Research Institute for Plant Preservation, University of Guelph, Guelph, ON N1G 2W1, Canada; monthona@uoguelph.ca (A.S.M.); spage01@uoguelph.ca (S.R.P.); mhesami@uoguelph.ca (M.H.)

**Keywords:** *Cannabis*, marijuana, marihuana, tissue culture, review, regeneration, floral reversion, micropropagation, TDZ, DKW

## Abstract

The recent legalization of *Cannabis sativa* L. in many regions has revealed a need for effective propagation and biotechnologies for the species. Micropropagation affords researchers and producers methods to rapidly propagate insect-/disease-/virus-free clonal plants and store germplasm and forms the basis for other biotechnologies. Despite this need, research in the area is limited due to the long history of prohibitions and restrictions. Existing literature has multiple limitations: many publications use hemp as a proxy for drug-type *Cannabis* when it is well established that there is significant genotype specificity; studies using drug-type cultivars are predominantly optimized using a single cultivar; most protocols have not been replicated by independent groups, and some attempts demonstrate a lack of reproducibility across genotypes. Due to culture decline and other problems, the multiplication phase of micropropagation (Stage 2) has not been fully developed in many reports. This review will provide a brief background on the history and botany of *Cannabis* as well as a comprehensive and critical summary of *Cannabis* tissue culture. Special attention will be paid to current challenges faced by researchers, the limitations of existing *Cannabis* micropropagation studies, and recent developments and future directions of *Cannabis* tissue culture technologies.

## 1. Introduction

*Cannabis sativa* L. is rising to prominence as a commercial crop for industrial, food, medical, and recreational applications. The current wave of interest has been characterized by a growing number of countries easing restrictions around research, commercial cultivation, and sale of dried *Cannabis* flowers, extracts, and consumable, medicinal, or industrial products. With interest renewed in this crop, which has been cultivated for thousands of years, research and innovation in the coming decades is expected to deepen our understanding of the growth, physiology, and biochemistry of *C. sativa.* Our improved understanding of this important plant will enable large-scale micropropagation, genetic preservation, and the development of plant biotechnologies for advanced new plant breeding technologies (NPBTs). The application of plant biotechnologies and NPBTs will require effective, high-throughput In Vitro culture systems that will allow for transformation and subsequent scale-up of any novel cultivars developed by micropropagation. Once developed, an effective transformation system will require regeneration and clonal propagation systems that can be reliably replicated in multiple lab environments and that can effectively be scaled up to meet the commercial industry’s needs. To build these robust regeneration and micropropagation systems, methods must be tested across multiple drug and fiber-type genotypes, be flexible with the age and condition of plant tissues, and be able to accommodate small differences in culture conditions that will inevitably arise from different lab environments and from the transfer to a large-scale commercial tissue culture operation. This review offers a critical analysis of the existing published and pre-print *C. sativa* micropropagation and regeneration literature and highlights the current shortfalls to help direct current and future research to catch up on decades of lost opportunities due to the criminalization and overregulation of *Cannabis.*

## 2. Brief History of *C. sativa* in North America

The relevance of *Cannabis* as a versatile crop for oilseed, fiber, medicinal, and recreational drug production spans millennia. Between 1000 and 2000 BCE, *Cannabis* was introduced to Western Asia, Europe, and Egypt as a fiber crop for producing cloth, ship ropes, and paper. After 500 CE, the cultivation of *Cannabis* was widespread across Europe [1,2]; however, it was not until 1545 and 1606 that it was introduced to South and North America, respectively [3]. Despite its centuries-long cultivation, the beginning of the 20th century saw its recreational use outlawed and medicinal use strongly curtailed by an addendum to the League of Nations’ 1912 Opium Convention. This act pushed countries around the globe to restrict and criminalize *Cannabis* [4]. 

In Canada, *Cannabis* was made illegal following its addition to the Opium and Drug Act in 1923 [5,6], and the United States followed suit with the 1937 Marijuana Tax Act, severely restricting the medicinal use of *Cannabis* in the United States [6,7]. *Cannabis* had been included in the United States Pharmacopoeia since 1850 and was removed in 1942, a few years after passage of the Marijuana Tax Act of 1937 [8]. In the United States, *Cannabis* is classified under the most restrictive drug class (Schedule I) as part of the Comprehensive Drug Abuse Prevention and Control Act of 1970. This 1970 act overturned the 1937 Marijuana Tax Act and states that *Cannabis* has “no apparent medical potential and a high likelihood of abuse” [9,10]. These restrictions, which made no distinction between fibrous hemp and drug-type *Cannabis,* had the unfortunate consequence of limiting most *Cannabis* research by making its acquisition for research purposes challenging [8,9]. Commercial production of industrial hemp (*C. sativa* with <0.3% Δ^9^-tetrahydrocannabinol (THC) by dry weight [11]) has faced many of the same restrictions as drug-type (>0.3% THC by dry weight) *Cannabis* in North America, as the distinction between the two has been largely ignored by government and law enforcement [9]. 

The strict conditions that regulate *Cannabis* research have created challenges throughout the research pipeline [10,12]. Early small-scale clinical trials have investigated the use of cannabinoids to treat comorbidities of autism spectrum disorder, anxiety, chronic pain, and seizures and have shown promising results, but research in this area has been highly restricted and progress has been slow [13,14,15,16,17]. Likewise, these restrictions and the lack of a legal industry have limited research on agronomic, horticultural, and biotechnological aspects of the crop. As a result, relative to the economic importance, technological development is in its infancy and many techniques that are routine for most species are not developed in *Cannabis*.

In recent years, this has started to change as countries around the world have started to lift some restrictions. In Canada, commercial production of hemp was legalized in 1998 [9]; however, regulatory barriers and a lack of market interest resulted in a very slow-growing industry until recently [18]. In the United States, a pilot-scale production of industrial hemp was legalized in 2014 followed by commercial-scale federal legalization in the 2018 farm bill [9,18]. Prior to this change, federally funded research in the US could only be conducted with *Cannabis* obtained from the National Institute on Drug Abuse (NIDA). With the passing of the 2018 farm bill, hemp can now be used for research, but drug-type *Cannabis* is still highly restricted at the federal level. In 2013, the Marihuana for Medical Purposes Regulations were implemented by the Government of Canada, laying the groundwork for commercial production of medicinal *Cannabis* [8,19]. The legalization of the possession, growth, and consumption of *Cannabis* for recreational purposes followed in October 2018. At the international level, regulations are also beginning to change; a landmark decision by the United Nations Commission on Narcotic Drugs (CND) voted to remove *Cannabis* from Schedule IV of the 1961 Single Convention on Narcotic Drugs in December 2020, thereby recognizing the medicinal and therapeutic uses of *Cannabis* [20]. While still highly regulated, the legalization of *Cannabis* for medical and recreational consumption in Canada, the legalization of hemp in the United States, and a similar trend around the world has resulted in a renaissance period for *Cannabis* research.

## 3. Botany and Taxonomy of *C. sativa*

**Figure 1:**
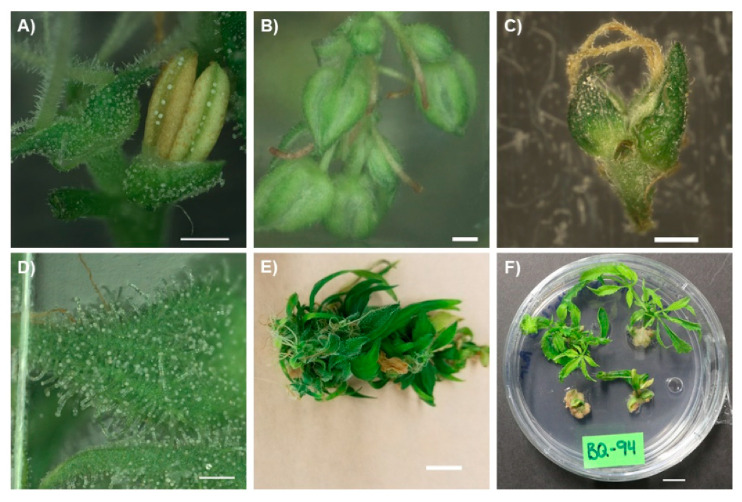
In Vitro flowering of *Cannabis sativa.* (**A**) Flowering *C. sativa* male plant displaying a hermaphroditic phenotype, showing female flowers (left) adjacent to male flowers (right). Scale bar—1 mm. (**B**) In Vitro male inflorescences of *C. sativa*. Scale bar—1 mm (**C**) A pair of female *C. sativa* florets obtained from In Vitro flowering *C. sativa*. Scale bar—1 mm. (**D**) Glandular trichomes developing on the bract surrounding the ovary of a female *C. sativa* inflorescence. Scale bar—2 mm. (**E**) Mature flowering In Vitro explant of *C. sativa.* Scale bar—1 cm. (**F**) Four-week-old vegetative explants reverted from In Vitro *C. sativa* inflorescences. Scale bar—1 cm.

*Cannabis sativa* L. (*Cannabis*, hemp, marijuana) is an annual flowering plant of the family Cannabaceae. Although *Cannabis* is usually dioecious, hermaphroditism occurs in some cultivars (Figure 1A) and both formal and informal breeding programs have resulted in some monoecious cultivars, primarily restricted to hemp [21,22,23]. The family Cannabaceae consists of ten genera, containing over 100 accepted species, with *Humulus lupulus* L. (hops; the chief ingredient in beer) being a notable member [24,25]*. C. sativa* is native to central Asia, likely in the foothills of the Himalayan Mountain Range [1,2]. *Cannabis* is a fast-growing plant, growing up to 10 cm a day and reaching heights of 6 m in its native habitat, while growth in temperate climates is usually lower [23,26,27].

**Figure 2:**
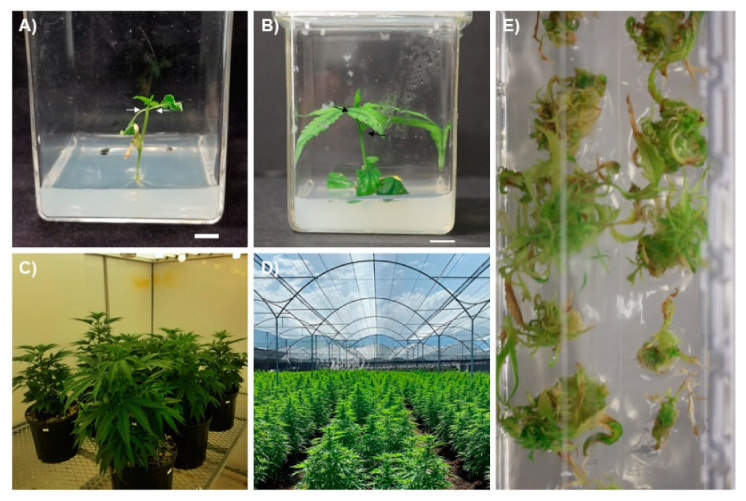
(**A**) In Vitro germinated seedling of *C. sativa* demonstrating opposite leaf arrangement. White arrows show oppositely oriented first true leaves. Scale bar—1 cm. (**B**) A Stage 2 vegetative explant (subcultured from a nodal explant) of *C. sativa* demonstrating alternate leaf arrangement (black arrows), a change in phyllotaxy resulting from explant maturation. Scale bar—1 cm. (**C**) *C. sativa* grown in controlled environment growth chambers under fluorescent lighting. (**D**) *C. sativa* grown outdoors under a shade cloth in Colombia. Image supplied courtesy of Avicanna™. (**E**) Hyperhydric *C. sativa* explants growing on Murashige and Skoog (MS) medium supplemented with 0.5 µM thidiazuron (TDZ).

When grown from a seed, the first true leaves are pairs of oppositely oriented single leaflets (Figure 2A). As the plant matures, the phyllotaxy shifts from opposite to alternate leaf arrangement and the number of leaflets per leaf increases (Figure 2B; Clarke 1999; Spitzer-Rimon et al. 2019). Leaves on a mature plant are digitate with anywhere from 5 to 11 leaflets and have a long petiole, although during flowering, they often revert to producing lower numbers of leaflets [23,28]. *Cannabis* is predominantly a short-day plant, with flowering induced by 12- to 14-h photoperiods [29]; however, some photoperiod-insensitive cultivars have been developed. Male and female plants cannot easily be distinguished until flowers begin to appear [26,30]. Male flowers have five green or yellow petals and are larger than female flowers (Figure 1B). Female flowers consist of an ovule enclosed in a thin green bract with two yellow/whiteish stigma emerging from the closed bracts (Figure 1C) [26,30]. During the development of the flower, before the elongation of the stigma, glandular trichomes develop on the bract surrounding the ovary (Figure 1D) [22]. Two main types of trichomes can be found covering *Cannabis* plants: glandular and non-glandular trichomes. Only the former produce cannabinoids in any considerable quantity, and glandular trichomes are predominantly found on the bracts and floral leaves of female plants (Figure 1D). Male plants produce few, if any, glandular trichomes [28,31]. Due to their low levels of cannabinoids, male plants are generally not consumed as a medicinal or recreational drug and will not be extensively discussed in this review.

The taxonomy of the genus *Cannabis* is a matter of spirited debate and no consensus has emerged on whether it is a monospecific or polyspecific genus [32,33,34]. The ability to distinguish between hemp and drug-type *Cannabis* has been the subject of much interest by law enforcement, which relies on THC content for distinction [35]. From a law enforcement and regulatory standpoint, the two main categories of *Cannabis* have been described as “drug-type” (medicinal or recreational) and “fiber-type” (industrial hemp), the drug-type generally being dioecious, with a short, wide, bush-like growth pattern, while the fiber-type can be either dioecious or monoecious with a tall and thin growth pattern [36]. However, this distinction is further complicated by hemp developed for seed or non-psychoactive cannabinoids, which often morphologically resemble drug-type *Cannabis*. Two distinct *Cannabis* chemotypes have been identified, which also fall in line with the two aforementioned morphological groups and are largely defined by their THC content. The fiber-type *Cannabis*, or “hemp”, has a THC dry weight in the flowering heads of <0.3% or <0.2% depending on the jurisdiction [9,11,18]. Hemp can often be accompanied by a higher cannabidiol (CBD) content (THC:CBD < 1), while the elite drug-type cultivars typically report a THC:CBD ratio >1, or >0.3% THC in the flower heads [9,37].

However, a taxonomic system based on THC:CBD ratios has faced scrutiny [38] and other classification systems that further divide the species based on chemotype have been suggested. These include classifications based on other secondary metabolites produced by the *Cannabis* rather than solely the THC and CBD levels [39,40]. Early genetic studies attempting to distinguish between the genetic fingerprints of hemp and *Cannabis* have suggested that the chief differentiation factor between the two plants was a single locus that determined the production of THC or CBD synthases [41]. These findings have been echoed by whole genomic and transcriptomic assemblies of hemp and drug-type *Cannabis,* which have shown that hemp plants have high levels of cannabidiolic acid synthase *(CBDAS*) genes and transcripts, while the *THCAS* gene encoding the oxidocyclase enzyme, which forms tetrahydrocannabinolic acid (THCA), is dominant in drug-type cultivars [2,42]. However, recent work using single-nucleotide polymorphisms (SNPs) has shown that the genetic differences between hemp extend beyond the loci responsible for cannabinoid production and are instead found throughout the entire genome [35].

Drug-type *Cannabis* has been historically described by enthusiasts as consisting of three species: *C. sativa*, *C. indica*, and *C. ruderalis*. The diversity of chemical and morphological traits within *Cannabis* has led some taxonomists to agree with this and propose that *Cannabis* should be considered a polyspecific genus containing multiple individual species: *sativa*, *indica*, and *ruderalis* [43,44,45]. Further sub-speciation has even been suggested within these groups [34,46]; however, this nomenclature has yet to be widely used. The taxonomy of drug-type *Cannabis* is complicated by years of prohibition, which resulted in informal, clandestine breeding programs that caused decades of interbreeding and hybridization without records of parentage [40,46]. The ability to consistently and reliably distinguish between *sativa* and *indica* types of *Cannabis* has been scrutinized [35], and as a result of these underground breeding programs, establishing the pedigree of *Cannabis* is incredibly challenging and has resulted in unpredictability for consumers of *C. sativa* products [33]. Concerns have also been raised that this ever-increasing introgression is leading to a decline in biodiversity in the species and a loss of native indigenous *C. sativa* varieties [46]. The ease of interbreeding within *Cannabis* has resulted in a highly polymorphic genome, which has led many researchers to classify *Cannabis sativa* as a monospecific, highly polymorphic species [32,47,48,49]. This debate is ongoing and has been reviewed extensively [32,34,45]. However, it is not the focus of this review and we will refer to species as presented by the authors when possible.

## 4. Current Production Practices

*Cannabis* is a highly adaptable species that can be grown in a variety of conditions, including outdoors in tropical or temperate climates or in controlled environments ranging from rudimentary greenhouse structures to sophisticated controlled environment facilities (Figure 2C,D) [36]. The production system of choice is determined based on the end-use of the plant. Plants grown to produce low-value commodities such as oilseed or fiber are typically cultivated exclusively outdoors, where production costs are low. In contrast, plants cultivated for dried flowers for recreational or medicinal use can be cultivated outdoors, in greenhouses, or in indoor production facilities. While production costs for recreational/medicinal products are also lower outdoors, there is a general belief that indoor production facilities produce higher-quality products, which justifies the extra costs for premium flowers [50]. However, with the growing trend toward extracts and purified cannabinoids, it is likely that much of the medicinal/recreational production (CBD from hemp, THC from drug-type *Cannabis*) will be done outdoors to capitalize on these lower production costs. The higher level of oversight offered in controlled environments also allows for easier management of insects and diseases, which is important in order to meet strict government health and safety regulations surrounding the use of chemical control agents, microbial load, and other quality assurance (QA) requirements [51,52]. These regulations have driven most of the commercial drug-type *Cannabis* production into greenhouses and indoor facilities for now [53].

As with production systems, the approach to plant propagation is influenced by the end-use of the plants. Traditionally, hemp has been cultivated by seed using large-scale, highly mechanized, production practices similar to other grain crops [54]. In contrast, drug/recreational *Cannabis* is generally propagated using clonal methods and treated as a horticultural crop [51,52,55]. This is done to mitigate the high level of phenotypic diversity displayed within seedling populations and to consistently produce high quality, uniform crops that meet consumer preferences and comply with government regulations [56]. While this variability also exists in hemp seed, the benefits of clonal propagation and manual planting do not justify the costs for oilseed or fiber [57]. However, new regulations surrounding the use of hemp to produce CBD and other non-psychoactive cannabinoids have led some hemp producers to use clonal propagation [18,54,57].

Clonal propagation can take many forms, but traditionally, *Cannabis* has been propagated through stem cuttings. In general, *Cannabis* is relatively easy to root, and large numbers of plants can be produced from a single mother plant [55]. While more expensive than seed, this approach can be efficiently used to mass-produce genetically and phenotypically uniform plants at a commercial scale to produce a more uniform crop. However, this approach requires the maintenance of mother plants in a vegetative state and can occupy 10–15% of the floor space in a commercial operation. The maintenance of mother plants also requires them to remain in a vegetative state. While this is easily accomplished for most genotypes, it presents challenges for day-neutral genotypes as they do not respond to photoperiod [29]. Perhaps of greatest importance, though, is that mother plants are susceptible to insects, pathogens, and viruses and can transmit these biotic factors to their cuttings and lead to problems during production. This is of importance in *Cannabis* as there are currently very few control options registered for the crop and there is a strong consumer preference for no pesticide use [18,51,52].

## 5. Micropropagation of *C. sativa*

**Figure 3:**
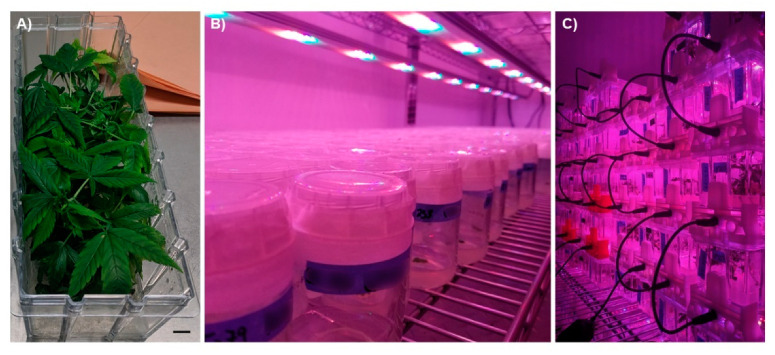
Healthy *C. sativa* explants growing in We-V boxes (**A**). Callus cultures growing in glass culture vessels under LED lighting in a controlled environment growth chamber (**B**) and high-density stackable culture vessels (We-V) with individually programmable LED lighting (**C**) demonstrate the variety and density with which *C. sativa* can be cultured under In Vitro conditions.

An alternative approach to clonal propagation that addresses many of the challenges of conventional *C. sativa* propagation is the use of micropropagation, which uses plant tissue culture to mass-propagate plants in a highly controlled environment using aseptic techniques. In micropropagation, plants are cultivated in culture vessels, typically in a multi-tier culture room or even in stackable vessels outfitted with light emitting diode (LED) lighting [58] (Figure 3). This allows large numbers of plants to be maintained in a very small space, thereby reducing the amount of floor space required to maintain mother plants. This is particularly attractive for producers that want to maintain a large genetic library but do not want to dedicate the amount of floor space that would be required otherwise. Tissue culture techniques also offer a variety of approaches that may help in maintaining day-neutral genotypes and for long-term genetic preservation. Most importantly, due to the sterile nature of plant tissue culture, it can be used to produce insect-/pathogen-/virus-free propagules to reduce biotic pressures.

The use of plant tissue culture for the propagation of disease-free plants has provided the foundation for clean plant programs in various crops since the late 1900s [59,60,61]. In some cases, certified disease-free plants produced through tissue culture are planted directly in the field for production, while in other cases, they are used as clean material that is further propagated through other means in highly sanitary conditions and tested for important diseases before being used for commercial production [62]. The latter model provides most of the benefits of micropropagation while reducing costs. This approach has been successful in the seed potato industry for developing a disease eradication system [59]. In the case of *Cannabis*, either approach could be taken, and the decision would need to be based on a careful analysis of the costs and benefits by the producer, which will include many factors such as the efficiency of micropropagation, labor costs, the value of additional floor space, risk assessment, and other factors.

**Figure 4:**
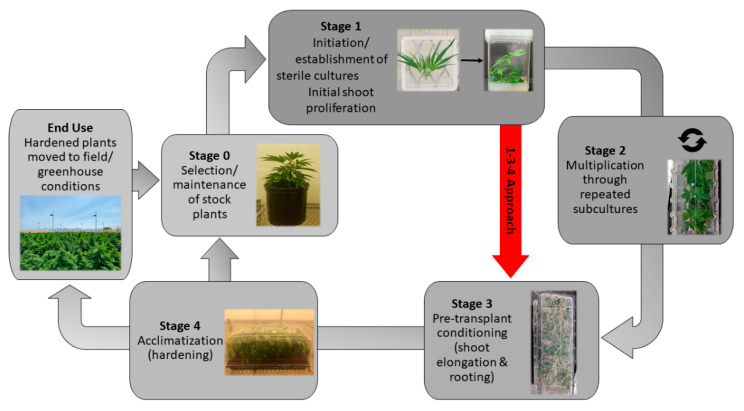
The five-stage micropropagation process for tissue culture. The red arrow is indicative of the 1-3-4 approach (where Stage 2 is skipped). Stage 2 is commonly skipped in *C. sativa* micropropagation methods due to the plant’s recalcitrance to long-term culture characterized by a slow decline in fitness. Inclusion of Stage 2 allows for repeated subcultures of In Vitro plants (indicated by the circular arrows), therefore facilitating large-scale multiplication or long-term germplasm storage.

The principal challenge in developing effective micropropagation methods is species and genotype specificity, resulting in many variations at each stage of micropropagation. Micropropagation is often broken down into five stages, where each stage needs to be optimized to establish a fully developed micropropagation method (Figure 4) [63,64]. These include Stage 0: Selection/maintenance of parent plant material; Stage 1: Initiation of cultures; Stage 2: Multiplication of shoots/embryos; Stage 3: Shoot elongation and rooting; Stage 4: Acclimatization (Figure 4). While the selection and maintenance of ex vitro stock plants are often ignored, the importance of stock plant health for the subsequent success of the cultures can have a significant impact on further results. Provided that the stock plants from Stage 0 are in good condition, the explants generally respond well to surface disinfection and produce an initial flush of growth during Stage 1. This initial flush of growth is often followed by a more sporadic growth pattern until the explants acclimatize to In Vitro conditions. It is in Stage 2, after plants acclimatize to In Vitro growth, where the largest benefit of micropropagation becomes apparent: the exponential multiplication of plants. Many horticultural crops are maintained for extended periods of time in Stage 2 and continuously sub-cultured for commercial-scale plant production. To illustrate the capability for rapid plant production, an In Vitro protocol using Stage 2 plants with a reasonable multiplication rate of 10 would produce one million plants after only six subcultures (10^6^). When a sufficient quantity of plants has been produced in Stage 2, they are then transferred to Stage 3 to elongate and develop roots, or alternatively, they are transferred directly from their In Vitro environment to an indoor growth facility/greenhouse to acclimatize, thereby combining Stages 3 and 4 (Figure 4). Combining these stages is often preferred for commercial applications as it reduces the number of steps In Vitro, thereby saving time and labor costs [65].

The earliest In Vitro studies of *Cannabis* were conducted in hemp and focused on determining its suitability for In Vitro culture and whether tissue culture would affect the agronomic and chemical characteristics of the plant (Table 1) [66,67,68,69]. Richez-Dumanois et al. [66] showed that hemp could be micropropagated using nodal cuttings and the inclusion of IBA and BAP promoted the growth of shoots from existing meristematic tissues. They also demonstrated the successful acclimatization of In Vitro grown hemp to greenhouse conditions [66]. Importantly, their work showed that the In Vitro grown plants’ chemical and physical profiles were similar to their greenhouse-grown counterparts. This finding has been reasserted by contemporary studies on medicinal *Cannabis* that found micropropagation from nodal cuttings had no significant effect on the cannabinoid contents of the mature flowering plant [69].

**Figure 5:**
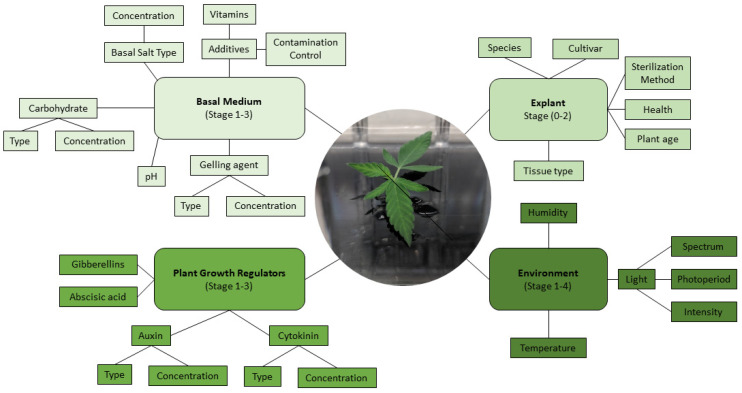
Tissue culture of healthy explants relies on the careful optimization of multiple factors. Center image: Freshly subcultured vegetative explant of *C. sativa*.

As it has been well established that *Cannabis* can be cultured In Vitro without affecting its biochemical outcomes, contemporary studies have shifted to determining the optimal growth and multiplication conditions for each stage of micropropagation, a task complicated by the numerous factors which must be considered when growing a plant In Vitro (Figure 5). Existing *Cannabis* micropropagation studies have primarily taken to optimizing freshly initiated tissues for shoot proliferation, opting to focus on plant growth regulator (PGR) combinations that result in rapid shoot proliferation (Table 1) [69,72,75]. Once developed, the shoots are rooted on an auxin-rich medium and then transferred back into growth facilities (Stages 3 and 4; Figure 4). These rapid and high-throughput approaches to *C. sativa* micropropagation are useful but neglect to study the long-term health and maintenance of the explants in culture, evidenced by the relatively few studies reporting results from Stage 2 (Table 1 and Table 2). The result of this is that explants are not fully acclimatized to In Vitro conditions, and consequentially, the protocols are only optimized for Stages 1, 3, and 4 (a 1-3-4 approach) rather than for the long-term conservation and multiplication of germplasm in Stage 2 (Figure 4 and Table 1).

It has been hypothesized that during Stage 1, explants have residual energy and endogenous plant growth regulators from the mother plants, resulting in an initial flush of growth during the initiation phase, followed by the sporadic growth response that has been observed until the cultures stabilize and acclimatize to In Vitro conditions [63,90]. Long-term culture decline of Stage 2 explants has been noted by Wróbel et al. [78], who reported a drop of 74–82% in the number of regenerated explants taken from Stage 1 plants. This initial flush of growth followed by a culture decline has also been observed by Page et al. [77], where previously published media compositions worked well for culture initiation (Stage 1), producing an initial flush of shoot proliferation, but failed to support long-term culture proliferation (Stage 2). Culture decline was manifested through high rates of hyperhydricity, callusing, and, in many cases, death of the cultures (Figure 2D) [77].

In these same studies, feminization of male plants cultured In Vitro on media optimized for Stage 1 growth was observed (Figure 1A). Feminization of *Cannabis* plants has previously been reported in male plants treated with ethylene, a plant growth regulator associated with the stress response in plants [91,92,93]. Reports of hermaphroditism in male *Cannabis* plants suggests the accumulation of ethylene in the culture vessels over time, likely as a response to less-than-optimal media to support Stage 2 growth. These findings highlight the current challenge of maintaining plants In Vitro long-term (Stage 2) using media from studies that have taken a 1-3-4 approach (Figure 4). There is a pressing need for further optimization of Stage 2 to develop a reliable five-stage micropropagation system in *Cannabis* and take full advantage of micropropagation.

Optimizing macro- and micronutrients for the culture of In Vitro plants represents one of the keystones to developing a successful micropropagation system (Figure 5) [94]. MS-based media are the current standard for *C. sativa* micropropagation studies (Table 1); however, few studies have conducted extensive comparisons between MS and other basal salts. Recently, a comparison of several basal salt mixtures (MS, Driver and Kuniyuki Walnut (DKW), B5, and BDS as modified at Arkansas Bioscience Institute (BABI)) by Page et al. [77] demonstrated better performance of Stage 2 explant growth using a DKW-based medium. On this medium, explants were healthier and had higher multiplication rates than their MS counterparts. DKW and MS are both relatively rich basal salts, suggesting that *Cannabis* requires high nutrient levels. The most notable difference between these two basal salts is that DKW contains higher levels of sulphur (~7×), calcium (~3×), and copper (10×) [77]. Interestingly, DKW was initially developed to address similar long-term declines in walnut cultures, comparable to what has been observed with *Cannabis* [95]. While Page et al. [77] demonstrated that DKW was more suitable for Stage 2 micropropagation of *Cannabis*, their findings likely represent an underestimate of the benefits, as the study was conducted for only one subculture of Stage 2 plants, while the issues of culture decline generally increase over multiple subcultures. Using DKW-based media, several cultivars of *Cannabis* have now been maintained for multiple years with no obvious signs of decline [89]. Despite the improvement over MS-based media, some signs of nutrient deficiency were still observed, and further improvements are possible through optimization of basal media.

Most protocols for micropropagation in *Cannabis* rely on shoot multiplication from existing meristems found in the apical and axillary nodes (Table 1). These meristems are regions of high cellular plasticity with cells early in their developmental state. Nodal cuttings can be grown In Vitro, much like vegetative greenhouse propagation. The most successful and frequently reported methods for In Vitro multiplication of *Cannabis* are those that rely on PGRs to cause shoot multiplication (SM) from a single nodal explant, resulting in the proliferation of shoots, often described as multiple shoot cultures (MSCs; Table 1). The highest yielding SM methods report between 9 and 13 explants per node [70,72] but used freshly initiated tissues from the greenhouse (Stage 1) and did not include an evaluation of Stage 2 performance. A review of the literature reveals a notable lack of *C. sativa* micropropagation studies using long-term In Vitro grown germplasms, or Stage 2, highlighting the need for future study in this area (Table 1).

In contrast to these reports of prolific MSCs, some authors have noted that *Cannabis* does not readily produce MSCs and instead tends to produce a single shoot with a high degree of apical dominance and low levels of branching, resulting in a much lower multiplication rate (Table 1) [75,78,80]. A recent study of two high-CBD *Cannabis* cultivars by Mestinšek Mubi et al. [80] found shoot multiplication between 0.59 and 1.78 on TDZ or meta-Topolin (mT) media recipes, which had previously been reported to yield between 11 and 13 shoots by other research groups [72]. Furthermore, the authors found that these media compositions did not significantly improve shoot proliferation over the PGR-free control (MS) in their two tested cultivars [80]. Similarly, Wróbel et al. [78] report that existing nodal propagation protocols result in multiplication rates of 0.9, resulting in a loss of plant material. Another alternative to traditional nodal shoot multiplication is the use of two node explants used by Page et al. [77]. Unfortunately, this alternative reduces the number of explants that can be obtained from a single plant, thereby limiting the overall multiplication rate.

To address this issue, a recent study proposed an alternative approach to *Cannabis* micropropagation in which single shoots are grown for a period of time and the apical meristem is removed in culture [78]. Removal of the apical shoot breaks apical dominance, allowing the axillary buds to develop into branches. Shoot tips from the developed axillary branches are then used as secondary explants for Stage 2 growth and multiplication, with the authors reporting a higher survival rate from shoot tips than nodal explants [78]. Using this approach, Wróbel et al. [78] increased the multiplication rate of Stage 2 explants from 0.9 when micropropagated on MS + 0.25 mg/L TDZ to 3.0 using this modified shoot tip micropropagation method on MS + 0.5 mg/L indole-3-acetic acid (IAA) medium. While this approach was effective and represents one of the few methods reporting multiplication of Stage 2 cultures (Table 1), the added step of removing the apical shoot requires more labor and increases the risk of contamination. It should also be noted that the explants were initiated into culture from seeds, and juvenile tissues generally respond better In Vitro than mature explants. However, the author reports that well-established subcultures were used in their experiments and that a minimum of ten culture cycles were performed before proceeding to Stage 3 (rooting). Another group reported that the addition of an auxin antagonist α-(2-oxo-2-phenylethyl)-1H-indole-3-acetic acid (PEO-IAA) could also contribute to breaking apical dominance and increase branching in seedling tissues, resulting in a multiplication coefficient of up to 1:10 [75]. This is a promising approach that merits further investigation.

### Floral Reversion: An Alternate Micropropagation Approach

As previously highlighted, some of the frequent challenges in *Cannabis* micropropagation include the lack of multiple shoot formation, a strong degree of apical dominance leading to low levels of branching, and poor survival of single-node explants. As a result, many studies using nodal tissues report low multiplication rates ranging from less than one (resulting in loss of stock plants) to about four explants per nodal culture (Table 1) [66,75,77,78,80]. From a practical standpoint, these multiplication rates are not suitable for many applications. In order to increase the multiplication rate through shoot proliferation, alternative approaches to increase the number of meristems per explant are needed. One potential approach is to induce flowering and use floral reversion. The inflorescence of the *Cannabis* plant is a highly branched compound racemose inflorescence that contains a large number of meristematic regions [22]. Initial observations found that some *Cannabis* plants initiate flower development In Vitro (Figure 1C,E), and recently, Moher et al. [29] demonstrated that flowering could be reliably induced using a short-day photoperiod, similar to what is observed in the field. As such, In Vitro flowering plants represent an alternative approach to increase the number of meristems per plant to potentially increase the multiplication rate.

The use of inflorescence tissues from *C. sativa* appears to be a promising alternative mode of micropropagation to nodal cultures [74,76], having been well studied in many species [62,96,97,98]. Inflorescences tissues that demonstrate the ability to return from a flowering phase of growth to a vegetative stage of growth are broadly described as undergoing floral or inflorescence reversion [62,96,97,98,99]. In Vitro PGR-induced floral reversion has been shown in a variety of dicots and monocots. In monocots, it is widely used in many commercially important crops such as grasses, palms, bananas, and grains [62,98,100,101,102]. In dicots, floral reversion has been employed less frequently; however, it has been shown in the Brassicaceae family and has been employed in conservation efforts of recalcitrant dicots [96,97,103,104].

In *C. sativa,* floral reversion has been studied in a very limited capacity. A recent publication from our lab provided the first known report of regeneration from floral explants of *Cannabis* [74]. Piunno et al. were able to show that In Vitro floral reversion was possible from two of the three commercially produced cultivars tested when using floral explants collected from greenhouse/indoor plants. This study was important as it demonstrated the ability of floral explants to produce phenotypically normal shoots but did not determine whether they were produced from existing meristems or through regeneration from non-meristematic tissues, or whether In Vitro plants could be used as a source of explants [74]. Subsequent work by Monthony et al. [76] shed light on the mechanism of floral reversion using flowering In Vitro *C. sativa*. Based on histological observations, it appears that the vegetative explants that reverted from floral tissues originated from existing meristems subtending the florets, similar to what has been reported in nodal cultures (Figure 1F). Survival was greater in explants that contained floret pairs rather than individual florets, and the estimated multiplication rate of 18.2 (Table 1) matched or exceeded protocols using nodal micropropagation [76]. While in-vitro-grown vegetative explants generally have 5–6 nodes, each flowering In Vitro plant produces approximately 24 florets, highlighting the potential to dramatically increase Stage 2 multiplication rates (Table 1) [76,77]. Furthermore, vegetative explants derived from florets under a long-day photoperiod could then be returned to short-day conditions to induce more In Vitro flowering. The re-flowering of reverted tissues provides a continuous micropropagation cycle consisting of flowering, reversion, vegetative growth, and re-flowering, which is ideally suited for Stage 2 growth. This alternative method may also provide a viable approach for the clonal propagation of day-neutral genotypes, which cannot be maintained in a continuous vegetative state of growth.

## 6. Regeneration in *C. sativa*

**Figure 6:**
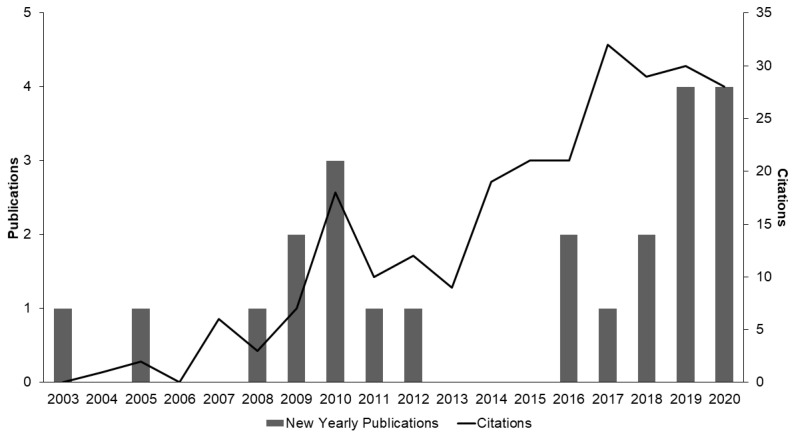
Yearly publications and the number of citations for articles matching the search TOPIC: (*Cannabis sativa* OR hemp) AND TOPIC: (regeneration) AND TOPIC: (micropropagation OR In Vitro OR tissue culture) on the Web of Science database. Data obtained from Web of Science® on 17 November 2020 using Microsoft Edge®. Data presented may not be a comprehensive representation of the available publications and are limited to publications indexed by Web of Science®.

While proliferation of explants from pre-existing meristems, such as the nodal propagation methods outlined in Table 1, typically results in low rates of mutation and good genetic fidelity, de novo regeneration systems (Table 2) can offer increased multiplication rates and are required for many other biotechnologies [105,106,107,108]. Vegetative nodes used for shoot proliferation represent a small fraction of the entire tissue composition of the plant and are ultimately limited. Regeneration from non-meristematic somatic tissues offers a larger pool of starting materials for micropropagation. As a result, de novo regeneration from somatic tissues through embryogenesis and organogenesis can greatly increase the number of explants produced in the same time-period. As the regulatory landscape evolves to facilitate research, interest in de novo regeneration of *Cannabis* has been increasing, yet the body of literature investigating regeneration systems in *Cannabis* remains limited (Figure 6; Table 2). A further complicating factor in the body of literature is the inconsistent use of the term regeneration, as some publications use it when referring to SM/MSCs from tissues containing existing meristems with no clear evidence of regeneration [67,70,74,78,82].

Earlier reviews on the state of *C. sativa* micropropagation and regeneration largely failed to underscore the many challenges in the existing body of research. These challenges include incomplete and ambiguously reported results [28,67,74,81,82,84,86], recalcitrance to regeneration [28,68,81,82,86,88]; genotype- and tissue-specific responses to regeneration [68,81,82,87,88]; and a lack of reproducibility of successful protocols in the literature [77,89].

### 6.1. Incomplete and Ambiguously Reported Results

Existing micropropagation protocols that rely on regeneration from non-meristematic tissues often report low levels of regeneration, and in some studies, the rate or frequency of germination is not reported, and ambiguous or no visual evidence is included (Table 2). Flores-Sanchez et al. [84], for example, reported the induction of somatic embryogenesis from *C. sativa* suspension cultures but they did not provide any data or visual evidence in support of their claims. Mandolino et al. [81] report “occasional” regeneration from hypocotyl tissues; however, they do not report the frequency of shoot production or the percentage of tissues that responded. Studies on callus cultures by Ślusarkiewicz-Jarzina et al. [82] state that across all treatments, they were able to achieve only 1.35% regeneration from callus cultures. In their study on somatic embryogenesis and organogenesis from stem and root tissues, Plawuszewski et al. [67] report successful regeneration in stem-derived callus but did not specify how much regeneration was achieved. A subsequent study published by this research group in 2008 included regeneration levels from stem tissues, reporting 14% regeneration in the most successful treatments [68]. Other authors have chosen to report callusing data, such as Farag [28] in their study of juvenile leaf callus. However, they do not specify the percentage of callus that regenerated, reporting only an average of 8.5 regenerants per callus. Movahedi et al. [86] also did not report the regeneration percentage, describing it as “low” with an average of less than one regenerant per seedling-derived callus culture. In a study on the regeneration potential of floral tissues, Piunno et al. [74] report organogenic regeneration from florets but do not specify the percentage of cultures that regenerated. They did not determine if this was de novo regeneration or proliferation of existing meristems but hypothesized that it was from pre-existing meristems and not de novo regeneration. As discussed above, the latter’s hypothesis has since been supported by histological examinations that identified the presence of quiescent vegetative meristems within *Cannabis* inflorescences, and the use of the term “regeneration” is inaccurate [76]. These studies highlight the value of more detailed reporting of results in regeneration experiments and showcase how, when reported, regeneration rates are often low (Table 2).

### 6.2. Genotype and Tissue Specificity

In tissue culture, the commitment to regeneration is highly dependent on the genotype, tissue type, and physiological state of the material. The range of genetic variability in *Cannabis* can be seen by the physiological and chemical differences between hemp and drug-type cultivars. Hemp produces negligible (<0.3%) levels of Δ^9^-tetrahydrocannabinol (THC; Government of Canada 2015) and has been bred for a high fiber and oil content [26]. In contrast, drug-type *Cannabis* can contain anywhere from 5% to over 20% THC and has primarily been bred for indoor production, highlighting the biochemical and morphological variability within the species [38]. This variability has also been demonstrated by the In Vitro responses of *Cannabis*, which show genotypic and tissue-dependent regeneration responses [68,81,82,87,88].

As previously discussed (see Section 2. Brief History of *C. sativa* in North America), access to *Cannabis* for research purposes has historically been limited. As such, most US-based federally funded research can only be conducted with *Cannabis* obtained from the NIDA [10,12]. Recently, concerns have been raised that NIDA-supplied medical-grade *Cannabis* is biochemically [109] and genetically homogenous and does not accurately represent the diversity available commercially [12]. Vergara et al. [109] showed that NIDA-supplied *Cannabis* has a limited cannabinoid profile and a lower concentration of THC compared to legal, dispensary-supplied *Cannabis* in the United States. Genetic evidence has also emerged which shows that NIDA-supplied *Cannabis* is more closely related to hemp than most drug-type genotypes, despite containing moderately high levels of THC [12]. Regeneration studies have been conducted more frequently and with more cultivars in hemp, and many of these studies have been more successful than studies using true drug-type cultivars [67,75,81,82,87,88]. The finding that NIDA-supplied *Cannabis* may be more genetically similar to hemp than commercially available drug types underscores the need to test existing regeneration studies on a large, representative sample of drug-type cultivars before findings from research using NIDA-supplied *Cannabis* can be assumed to have general applicability.

The regenerative potential of tissues varies from species to species and is usually determined empirically [110]. One of the earliest studies on regeneration was conducted on four tissue types (leaf, hypocotyl, cotyledon, and roots) in 12 commercial hemp cultivars. In this study, Mandolino et al. [81] reported that callus formation from somatic tissues was achieved in all 12 cultivars across all four tissues; however, callus morphology varied, ranging from small amounts of brown callus to high amounts of white friable callus depending on the cultivar and tissue tested. Despite the formation of callus in all cultivars, only “occasional” regeneration from callus was obtained in one of the 12 tested cultivars. Mandolino’s qualitative analysis of regeneration found that it was uneven across the tested tissues and that hypocotyls were the most likely, and roots the least likely, to respond. No regeneration was obtained from leaf tissues [81].

Following this early study by Mandolino et al. [81], subsequent studies tried to identify which tissues in *Cannabis* are best suited to regeneration; however, no clear consensus has emerged. Ślusarkiewicz-Jarzina et al. [82] assessed regeneration of young leaves, petioles, internodes, and axillary buds in five hemp cultivars and found that callusing and regeneration levels were cultivar- and tissue-dependent. The authors found that petiole and leaf tissues were most responsive to regeneration; however, the magnitude of this response varied among cultivars. In petioles, callusing ranged from 27% to 83% depending on the cultivar tested [82]. Total regeneration by cultivar was low and ranged from 0% to 6% [82]. Wielgus et al. [68] also reported a cultivar-specific response in their study on direct organogenesis in three hemp cultivars. Of the three cultivars tested, two showed a less than 2% regeneration response, and in the stem tissues of the third cultivar, only 14% underwent direct organogenesis [68]. The study also compared the response amongst three types of seedling-derived tissues: cotyledons, stems, and roots. Wielgus and colleagues found that “seedling stem tissue” (assumed to be hypocotyl tissue) was most responsive and cotyledon tissues were least responsive. Using seedling-derived leaf tissues, Farag [28] reported successful callogenesis and subsequent regeneration with an average of 8.5 regenerants per callus; however, the % regeneration was not stated.

A 2020 study by Galán-Ávila et al. on direct organogenesis of hypocotyl, cotyledon, and true leaves in five hemp cultivars also found that hypocotyls were most responsive across all five cultivars. In their study, 49.5% of hypocotyl tissues responded across all treatments, compared with only 4.7% of cotyledon and 0.42% of true leaves [88]. The regeneration response was tissue- and cultivar-dependent, ranging from 2% to 71% response depending on the source tissue and cultivar [88]; however, within hypocotyl treatments, this response range showed less variability (32–71% regeneration). The number of shoots produced per explant was consistently between one and two [88]. The findings that hypocotyl tissue is highly favorable for regeneration echo the early *Cannabis* micropropagation studies by Mandolino et al. [81]. While Wielgus and Galán-Ávila both reported low regenerative capacity in cotyledons, a study by Chaohua et al. [87] has reported differing results. In their study, Chaohua et al. [87] assessed the efficacy of regeneration in 1- to 6-day-old cotyledon tissues from eight commercial hemp cultivars. While regeneration varied from 35.7% to 54.8% depending on the cultivar, these reported results highlight the regeneration potential of hypocotyl tissues and the contradictory nature of the existing studies on *Cannabis* regeneration with respect to tissue source [87].

In contrast to the several aforementioned reports on hemp, only one study examining regeneration responses (Table 2) across multiple drug-type genotypes has been published to date. This study by Piunno et al. [74] assessed the potential of floral tissue as a genesis for explant regeneration in three commercial medicinal *Cannabis* genotypes. Their publication reported that regeneration was only observed in only two of the three tested genotypes and at “low levels” (which were not further specified) [74]. Further research has suggested that this was not likely de novo regeneration [76], highlighting the absence of studies on the de novo regeneration of drug-type *Cannabis*.

### 6.3. Recalcitrance to Regeneration

Recalcitrance to regeneration has been reported throughout the existing literature with very few exceptions. Recent *Cannabis* regeneration studies have struggled to obtain high regeneration, shoot proliferation, and response rates. This challenge has already been overcome in many species and overcoming it is a prerequisite for the effective use of regeneration systems in many areas of plant biotechnology. In a regeneration study of five hemp cultivars, Galán-Ávila et al. [88] found that only 54% of hypocotyl tissues underwent organogenesis, reporting an average of 1.49 shoots/hypocotyl. A study of epicotyl tissues conducted by Movahedi et al. [86] reported similarly low rates of shoot multiplication of ~2 shoots per epicotyl, and the percent response was not specified. In a regeneration study using juvenile leaves, Farag [28] also neglected to report the % of callus cultures that underwent regeneration, reporting only a shoot proliferation of 8.5 shoots/callus culture. Chaohua et al. [87] found that across cultivars, 46.7% of 3-day-old cotyledon tissues underwent regeneration on MS media supplemented with TDZ and NAA, and the most responsive of the eight tested cultivars produced three shoots/explant. The media used by this author resemble media used by Lata et al. [85]; however, the media used differed in the levels of TDZ and NAA and the starting tissues were different, highlighting the importance of tissue type and medium composition. These differences are likely the reason that Chaohua et al. [87] reports lower regeneration rates than those reported by Lata et al. [85] (3 vs. 12.3 shoots/explant, respectively). Medium composition has been highlighted as a factor that could affect recalcitrance in *Cannabis* tissue culture by Page et al. [77]. In this study, the authors found that commonly favored MS salts supplemented with TDZ resulted in high levels of hyperhydricity (Figure 2E), poor explant development, and occasional death in five commercially available cultivars of drug-type *Cannabis* [77]. These phenotypes were largely reversed when the explants were cultured on media using DKW basal salts rather than MS [77]. Future studies hoping to develop a robust and replicable regeneration methodology are needed to determine the extent to which recalcitrance is a species-wide phenomenon and identify genotypes of *Cannabis* that are amenable to regeneration.

### 6.4. Lack of Reproducibility

Reproducibility of existing methods remains a challenge in both regeneration [89] and SM systems [80]. As previously discussed, with most contemporary US-based studies presenting data from a single cultivar [28,78,85,86], it is likely that this lack of genotypic diversity is contributing to the struggle with protocol reproducibility in *Cannabis* tissue cultures. The lack of diversity in drug-type *Cannabis* studies is juxtaposed with regeneration studies on commercial hemp varieties which frequently use between 5 and 12 cultivars, offering findings that are more representative of the general biological responses across industrial hemp cultivars rather than a single specific cultivar (Table 1) [81,82,87,88].

While many studies report signs of recalcitrance to regeneration, this is not universal. The most successful report of regeneration from somatic tissues of medicinal *Cannabis* was published in 2010 by Lata et al., in which they reported indirect regeneration in 96.6% of callus derived from young leaf material, with an average of 12.3 shoots per culture. Callus induction was achieved on MS media with 0.5 μM NAA and 1.0 μM TDZ followed by a transfer to MS media with 0.5 μM TDZ, which induced the high levels of regeneration they reported [85]. This protocol reported unprecedentedly high levels of de novo regeneration, making it an optimal protocol to be used in the application of plant biotechnologies. Until recently, this decade-old protocol had not been replicated in any publication by an independent research group or used for subsequent biotechnological development by the original authors. A 2020 replication study testing this protocol across 10 simple sequence repeats (SSR) characterized *Cannabis* cultivars found that callus was formed but no regeneration was observed [89]. The contradictory findings between these two studies raise concerns about the applicability of existing tissue culture methods across genotypes and/or their reliability.

Strong cultivar-specific responses to treatments have been noted in *Cannabis* tissue culture and this likely contributes to the lack of reproducibility in both shoot proliferation and regeneration-based systems [55,74,77,79,87,89]. As *Cannabis* research becomes more accessible, the implications of drawing sweeping conclusions based on single-cultivar studies have come under scrutiny [12,89], and evidence has called into question the genotype-independent responses implicit in many of these single-cultivar studies [77]. It will be the goal of future studies to establish complete and detailed methodologies applied across a broad genotypic sample to overcome recalcitrance in the species or at least to identify amenable cultivars to overcome the challenges currently faced in the tissue culture of *Cannabis*.

## 7. Genetic Stability and Preservation

One of the main applications of micropropagation is the preservation of genetics in a safe environment, free from biotic pressure. Tissue sources used for micropropagation carry varying probabilities of experiencing mutations, referred to as somaclonal variation, which can be problematic for the maintenance of clonal lines of plants [111]. Micropropagation through the proliferation of existing meristems is generally considered to have a lower mutation load than the use of de novo regeneration, especially for indirect de novo regeneration in which there is a callusing phase [111,112]. As such, shoot proliferation is often preferred for genetic preservation, although there is still a risk of somaclonal variation occurring [112].

In general, micropropagated *Cannabis* has been shown to produce plants that are morphologically and chemically similar to the parent material [69]. Furthermore, authors have reported that it appears to be genetically stable, with a low occurrence of mutations [56,69,72,75,113]. However, most studies that have assessed the genetic fidelity of micropropagated *Cannabis* did not assess it over long-term maintenance with many subcultures. Additionally, they used specific genetic markers such as Inter Simple Sequence Repeats (ISSR), resulting in only the detection of mutations that occurred at those specific sequences, which does not quantify the mutation rate across the whole genome [113]. As such, the actual mutation rate in *Cannabis* plants, including both In Vitro and ex vitro, has not been reported and there is significant potential for mutations to occur in both systems. Further research using more advanced sequencing techniques is needed to determine the relative mutation rate of plants growing in the greenhouse/indoors/outdoors, micropropagated plants produced through shoot proliferation, and plants produced through de novo regeneration.

While the actual mutation rate in these various settings has not been quantified, several approaches are known to mitigate somaclonal variation, some of which have been explored in *Cannabis*. The first approach is using low-temperature cultures in which plant growth is slowed down by maintaining them at lower-than-normal temperatures. This was first reported by Lata et al. [113], who encapsulated axillary buds and were able to store them for 6 months in 5 °C storage before planting. The ISSR profiles of the cold-stored plants were comparable to the mother plant, making this approach suitable for maintaining genetic lines [113]. A logical extension of this is the use of cryopreservation to store tissues at cryogenic temperatures. Since this process essentially halts plant metabolism and cell division, it can be used to store plant genetics indefinitely and eliminate the accumulation of genetic mutations during the storage period [114,115].

An important aspect of genetic preservation is to recognize that all plants mutate as they grow, including plants in ex vitro conditions, making cryopreservation one of the only approaches to prevent mutations from occurring in clonal lines over time [116]. While cryopreservation requires a significant up-front cost and more sophisticated facilities, studies in other species have demonstrated that it is often more cost-effective than in situ preservation over the long term [117]. As such, cryopreservation represents the most effective approach for maintaining genetic fidelity of clonal lines over long periods and may be economically advantageous. Uchendu et al. [118] have begun the development of a cryopreservation protocol for *Cannabis* by testing different plant vitrification solutions on shoot tips. Their highest regrowth rate was 63%, providing a viable approach for *Cannabis* germplasm conservation.

## 8. Future Directions

As highlighted in Table 1 and Table 2, most *Cannabis* micropropagation studies have focused on evaluating the effects that PGRs and explant type have on regeneration. However, other factors that affect the efficiency of regeneration protocols are often neglected. In this section, we highlight these overlooked factors that affect In Vitro *Cannabis* propagation and present promising new approaches studied in other species that could be used to overcome the hurdles currently faced in *Cannabis* research. As stated previously, genetics is one of the key factors influencing the regenerative capacity of a cultivar. This genotypic effect is well documented in the micropropagation of *Cannabis* and is, in part, due to the differences in endogenous phytohormones’ concentrations specific to individual cultivars [119]. Unfortunately, understanding of the biochemistry and metabolism of *Cannabis* beyond the cannabinoid pathways is limited and represents a necessary area of future study. To date, several studies [120,121,122,123,124] showed that the gene expression pattern of endogenous PGRs and the balance between endogenous and exogenous PGRs play an important role in regeneration efficiency, especially in recalcitrant plants. For instance, Kumari et al. [120] studied the endogenous level of PGRs via UHPLC–MS analysis and its effect on shoot regeneration and somatic embryogenesis of *Tulbaghia simmleri*. They reported that endogenous phytohormone signaling and transport affects In Vitro biological processes related to auxin and cytokinin, and generally, high-frequency regeneration protocols can be achieved when there is a balance between endogenous and exogenous PGRs. A similar approach was recently taken by Smýkalová et al. [75], who carried out UPLC-MS-guided studies on the effects of exogenous application of auxin, cytokinins, and their inhibitors in *Cannabis*. They showed that ex vitro hypocotyl segments have insignificant endogenous concentrations of aromatic and free forms of cytokinins but have high concentrations of O-glucoside and riboside bases of endogenous cytokinins. These studies have highlighted strong apical dominance in the species and represent a forward-thinking model for future studies. The continued development of such biochemical and molecular studies will prove imperative to overcoming recalcitrance of *Cannabis* to In Vitro regeneration.

The physiological condition of the mother plant and the type, position, size, and orientation of the explant play a pivotal role in micropropagation [125]. The source of the explant (i.e., ex vitro and In Vitro) also has an impact on regeneration [126]. Generally, In Vitro explants have more regeneration potential than ex vitro explants due to their juvenility and since they are already adjusted to In Vitro conditions [127]. There are, however, no studies comparing the regeneration potential of ex vitro and In Vitro *Cannabis* explants. The type of explant (e.g., cotyledon, leaf, node, root, etc.) also impacts the plant’s ability to regenerate. This is mainly due to differences in their endogenous phytohormone levels. Another overlooked factor is explant orientation, which can affect the initiation site, polarity, and regeneration efficiency [127,128]. Generally, horizontally positioned explants have higher regeneration rates than vertically positioned explants. This is likely due to the explants having more surface area in contact with the medium. For instance, Jun-jie et al. [128] reported that leaf segments oriented abaxially (lower surface facing down) had significantly higher shoot production than those facing upwards (adaxial). However, there are no reports in *Cannabis* that compare explant type, age, and orientation in In Vitro propagation. These studies will help clarify how these factors can be optimized to improve *Cannabis* regeneration protocols.

The incubation conditions, especially light and temperature, play an important role in regeneration efficiency. Wavelength, photoperiod, and flux density have a significant impact on In Vitro morphogenesis, photosynthesis, and phototropism [129] and require further study in *Cannabis*. Different species have different responses to light conditions [129,130]. Some plants may respond positively to the addition of photosynthetic photon flux, particularly under mixotrophic/photoautotrophic conditions (CO_2_-rich and low sugar level). Temperature also affects different biological processes such as photosynthesis and respiration [131]. Although the growth chamber temperature commonly ranges from 20 to 27 °C, the optimal temperature can vary based on the genotype. Despite the importance of light and temperature conditions, there are no studies on the effects these conditions have on in-vitro-grown *Cannabis*. It is essential to optimize these conditions to improve *Cannabis* micropropagation.

The composition of the culture medium, including gelling agents, carbohydrates, additives (e.g., PGRs, activated charcoal, phloroglucinol, and nanoparticles), basal salts, and vitamins, is the most important element of a tissue culture protocol and is often the focus of micropropagation and regeneration studies, including those previously discussed (Table 1 and Table 2). Generally, a culture medium can be categorized as a semi-solid or liquid medium. Although the concentration of gelling agents plays a conspicuous role in regeneration efficiency [132], there are no reports for *Cannabis* comparing the different types and concentrations of gelling agents. Carbohydrates are essential for many species in culture and there are many different sources (sucrose, glucose, fructose, maltose, glycerol, etc.). Different cultivars or species may react differently depending on the carbohydrate source, and some sources can be used for certain In Vitro processes depending on their roles in metabolism [133]. No existing *Cannabis* micropropagation and regeneration studies have compared the effects of carbohydrate sources on regeneration or explant growth. Currently, sucrose remains the carbohydrate source of choice when preparing media for In Vitro studies of *Cannabis* [70,77,78,80,89]. Hence, it seems that studying the effects of carbohydrate sources on *Cannabis* micropropagation may be an avenue for improving available In Vitro regeneration systems.

Adjusting PGRs and additives, especially balancing auxins and cytokinins, is a common experiment in plant tissue culture systems because the auxin/cytokinin ratio is often required for callogenesis, organogenesis, embryogenesis, and rhizogenesis. Most of the *Cannabis* micropropagation studies have investigated the effects of common auxins (e.g., 2,4-D, NAA, IBA, and IAA) and cytokinins (e.g., BAP, Kinetin, mT, and TDZ) on In Vitro regeneration (see Table 2). However, there are some lesser-used additives such as nitric oxide (NO) [134], polyamines [135], and nanoparticles [136] that have shown promising outcomes in other plant species. NO is categorized as a new phytohormone that plays a pivotal role in different biological processes, especially in cell division [137], morphogenesis [138], organogenesis, rhizogenesis [139], and plant defense mechanisms [134]. Several studies [140,141,142,143] have recently demonstrated the positive role of exogenous NO and/or sodium nitroprusside on callogenesis, shoot regeneration, and root initiation in different plants. Other studies [144,145,146] have shown that polyamines act as a key signal in organogenesis and shoot regeneration. In addition, nanoparticles (e.g., TiO_2_, Ag, Zn, ZnO, graphite, graphene, carbon nanotubes, quantum dots, and polymer dendrimers) have been successfully used in different In Vitro processes from the first step, through decontamination, to callogenesis, organogenesis, somatic embryogenesis, shoot regeneration, the study and production of secondary metabolites, and somaclonal variation [147,148,149,150]. For instance, Sarmast et al. [151] showed that adding 60 mg/l Ag nanoparticles to MS medium containing 0.1 mg/l IAA and 2.5 mg/l BAP increased shoot regeneration frequency, shoot number, and length of *Tecomella undulata*. Generally, the positive impact of nanoparticles in plant tissue cultures might be due to their role in delaying senescence through downregulation of genes such as aminocyclopropane-1-carboxylic acid synthase ACS gene [151] and microRNAs such as miR408 and miR398, related to ethylene production [152], and by decreasing the content of proline, hydrogen peroxide, and malondialdehyde through the improvement of antioxidant enzymes’ activity [153]. While micropropagation of *Cannabis* is a relatively new area in the established field of tissue culture, recent advances in nanotechnology and phytohormone signaling could be readily applied to the field of *Cannabis* tissue culture.

Vitamins and basal salts are the main components of medium compositions. MS basal medium has been widely used for *Cannabis* micropropagation. Recently, Page et al. [77] demonstrated that nodal explants of *Cannabis* cultivated on MS medium resulted in abnormal morphology and that the DKW medium was better at supporting shoot growth and promoting callogenesis in leaf disk cultures. However, Page et al. [77] also reported that the explants cultured on DKW medium still had some physiological defects, suggesting that further optimization is needed. Formulating and optimizing a new basal medium is time-consuming and expensive due to the number of essential elements and vitamins and their interactions that need to be considered. To solve this challenge, machine learning algorithms as a statistical and computational approach have been suggested as a solution [154]. Recently, machine learning algorithms have been used for developing and optimizing medium in multiple species such as *Pistacia vera* [155], *Centella asiatica* [156], pear rootstock [157], and chrysanthemum [158]. Therefore, these methods can be used for designing and optimizing *Cannabis*-specific medium and environmental conditions.

As highlighted in this review, micropropagation is a complex, multi-variable, and non-linear process that can be influenced by many factors, especially incubation conditions, medium composition, explant, genotype, and their interactions (Figure 5). The application of new computational approaches such as machine learning algorithms for analyzing, predicting, and optimizing In Vitro processes represents a forward-looking and novel approach to solving the challenges faced in *Cannabis* tissue culture. Recently, different machine learning algorithms have been successfully used for predicting and optimizing different micropropagation systems such as shoot regeneration, embryogenesis, androgenesis, and rhizogenesis, underscoring that these in silico predictions are viable and effective In Vitro [154]. Advances in machine learning, the synthesis of novel PGRs, and the application of cutting-edge nanoparticle technologies to tissue culture can open a new window for the comprehensive study of *Cannabis* micropropagation and pave the way to tackling the persistent recalcitrance and poor replicability affecting current In Vitro tissue culture studies of *C. sativa*.

## 9. Conclusions

The body of *Cannabis* micropropagation and regeneration literature is poised to undergo substantial growth as regulations around the globe begin to relax. While several existing publications report high rates of MSCs, there have been challenges in replicating the results of these studies across genotypes and research groups. Reports of de novo regeneration are even more limited; their success has been mixed and positive outcomes have been difficult to replicate. These challenges are highlighted by the fact that there are no published reports of regeneration of transgenic plants obtained using traditional molecular and genome editing approaches. In drug-type *Cannabis*, micropropagation and regeneration protocols suffer from low multiplication rates, poor replicability, and a vast array of starting tissues to choose from, coupled with high diversity in genotypic responses and underwhelming robustness resulting from protocols conceived using single genotypes. Precise methods using multiple genotypes are necessary to develop protocols that can be reliably replicated by other research groups, and innovative new approaches to *Cannabis* micropropagation are required if developments in *Cannabis* tissue culture and plant biotechnologies are to keep pace with the needs of the producers and consumers in this burgeoning industry.

## Figures and Tables

**Table 1:** Summary of the published micropropagation studies on *C. sativa.* The table summarizes studies that rely on shoot multiplication (SM) to increase explant number. SM refers to the proliferation of multiple shoots from an existing meristem, such as axillary or apical nodes and floral meristems. *Cannabis* type is defined in this table as either psychoactive “drug-type” (*Cannabis*; tetrahydrocannabinol (THC) > 0.3% in flowering head) or known industrial hemp genotypes “fiber-type” (Hemp; THC < 0.3% in flowering head). A breakdown of the cultivars (CVs) used in the study and the number which responded to the treatment are included for each study. N.S. Not specified is assigned to data that were not specified, instances of “data not shown”, or when data are omitted in the original research article.

Source	Explant (Response)	*C. sativa* Type (#CVs Responded/Used)	Best Media	Best Results	Stages Reported
Richez-Dumanois et al., 1986 [66]	Apical and axillary nodes (SM)	Fiber-type (2/2)	**SM:** MS + 0.5 μM BAP + 0.1 μM IBA	**SM:** 2 shoots/explant (apical meristem), % response N.S.	Stage 0: Y Stage 1: Y Stage 2: N Stage 3: Y Stage 4: Y
			**Rooting:** MS + 0.2% activated charcoal + 10 μM IBA	**Rooting:** 47.7% response	
Lata et al., 2009a [70]	Axillary nodes (SM and rooting)	Drug-type (1/1)	**SM:** MS + 0.5 μM TDZ	**SM:** 12.6 shoots/explant 100% response	Stage 0: Y Stage 1: Y Stage 2: N Stage 3: Y Stage 4: Y
			**Rooting:** ½ MS + 2.5 μM IBA + 0.05% activated charcoal	**Rooting:** 4.8 roots/explant 95% response	
Lata et al., 2009b [71]	Alginate encapsulated axillary nodes (Shoot induction and rooting)	Drug-type (1/1)	**Shoot induction:** MS + 0.5 μM TDZ + 0.075% PPM	**Shoot induction:** 11.8 shoots/explant (90 days; avg. 30 explants)	Stage 0: Y Stage 1: Y Stage 2: Y Stage 3: Y Stage 4: Y
			**Rooting:** (1:1) sterile fertilome: coco natural growth medium + MS + 0.5% PPM	**Rooting:** 100% conversion from encapsulation (90 days)	
Lata et al., 2016 [72]	Axillary nodes (SM and rooting)	Drug-type (1/1)	**SM:** MS + 2 μM mT	**SM:** 13.4 shoots/explant 100% response	Stage 0: Y Stage 1: Y Stage 2: N Stage 3: Y Stage 4: Y
			**Rooting:** MS + 2 μM mT	**Rooting:** 13.8 roots/explant 96% response	
Grulichova et al., 2017 [73]	Shoot tips (SM)	Fiber-type (2/2)	**SM:** MS + 0.54 μM NAA + 1.78 μM BAP ^a^	**SM:** Shoots/explant N.S. % response N.S.	Stage 0: Y Stage 1: Y Stage 2: N Stage 3: N Stage 4: N
Piunno et al., 2019 [74]	Immature and mature inflorescences (shoot induction and rooting)	Drug-type (2/3)	**Shoot induction:** MS + 10 µM TDZ	**Shoot induction:** 4 shoots/floral cluster % response N.S.	Stage 0: Y Stage 1: Y Stage 2: N Stage 3: Y Stage 4: Y
			**Rooting:** MS + 1.86 µM kinetin + 0.54 µM NAA ^a^	**Rooting:** Describes ‘most’ cultures as rooting.	
Smýkalová et al., 2019 [75]	Shoot apex, isolated apical meristem, and cotyledonary nodes from seedlings (SM, shoot development, and rooting)	Fiber-type (1/1)	**SM:** IMB4 + 6.97 µM KIN + 0.81 µM BAP9THP + 0.11 mM adenine hemisulphate ^a^	**SM:** 4.4 shoots/explant (isolated meristems) ~96% response	Stage 0: Y Stage 1: Y Stage 2: N Stage 3: Y Stage 4: N
			**Shoot development:** ½ MS no PGRs	**Shoot development:** N.S.	
			**Rooting:** ½ MS + 0.20 µM NAA ^a^	**Rooting:** 50% response	
Monthony et al., 2020a [76]	Single and pairs of florets (floral reversion and rooting)	Drug-type (2/2)	**Floral reversion:** DKW w/vitamins + 1 µM mT	**Floral reversion:** Estimated 18.2 explants derived from one In Vitro flowering plant 81% response	Stage 0: N Stage 1: N Stage 2: Y Stage 3: Y Stage 4: Y
			**Rooting:** DKW w/vitamins	**Rooting:** 44% rooted	
Page et al., 2020 [77]	Axillary nodes (SM)	Drug-type (4/5)	**SM:** DKW + 0.5 μM TDZ	**SM:** 2.23 shoots/explant 80% response	Stage 0: N Stage 1: N Stage 2: Y Stage 3: N Stage 4: N
Wróbel et al., 2020 [78]	Shoot tips and nodes from axillary branches (SM and rooting)	Fiber-type (1/1)	**SM:** ½ MS + 2.85 μM IAA ^a^	**SM:** 2.5 shoots/explant 70% response	Stage 0: Y Stage 1: Y Stage 2: Y Stage 3: Y Stage 4: Y
			**Rooting:** ½ MS + 2.85 μM IAA ^a^	**Rooting:** 74.6% rooted	
Codesido et al., 2020 [79]	Axillary nodes (SM)	Drug-type (6/6)	**SM:** Formula βH media	**SM:** Shoots/explant N.S. 58% response	Stage 0: Y Stage 1: Y Stage 2: N Stage 3: N Stage 4: N
Mestinšek Mubi et al., 2020 [80]	Axillary nodes (SM)	Drug-type ^b^ (2/2)	**SM:** MS+ 2.07 µM mT ^a^	**SM:** 1.78 shoots/explant 97.8% response	Stage 0: Y Stage 1: Y Stage 2: N Stage 3: Y Stage 4: Y
			**Rooting:** MS + no PGRs	**Rooting:** % response N.S.	

^a^ Molarity values converted from mg/L. ^b^ Authors reported using a high-cannabidiol (CBD) drug-type *C. sativa*, % THC not specified.

**Table 2:** Summary of the published regeneration studies on *C. sativa*. The table summarizes studies that rely on shoot regeneration for the production of *C. sativa* explants. Shoot regeneration refers to the formation of de novo shoots from non-meristematic tissues such as leaves, stems, or cotyledons. This includes direct and indirect organogenesis and somatic embryogenesis from callus or suspension cultures. *Cannabis* type is defined in this table as either psychoactive “drug-type” (*Cannabis*; THC > 0.3% in flowering head) or known industrial hemp genotypes “fiber-type” (Hemp; THC < 0.3% in flowering head). A breakdown of the cultivars (CVs) used in the study and the number which responded to the treatment are included for each study. N.S. Not specified is assigned to data that were not specified, instances of “data not shown”, or when data are omitted in the original research article.

Source	Explant (Response)	*C. sativa* Type (#CVs Responded/ Used)	Optimal Media	Optimal Results	Stages Reported
Mandolino and Ranalli, 1999 [81]	Leaf, hypocotyl, cotyledon, and root (Callogenesis and shoot regeneration)	Fiber-type (1/12)	**Callogenesis/** **shoot regeneration:** MS + B5 vitamins + 13.57–45.24 µM 2,4-D + 0.04–0.44 µM BAPa	**Callogenesis/shoot regeneration:** One tested cultivar occasionally gave rise to organogenic callus from hypocotyl tissue. % regeneration N.S.	Stage 0: N Stage 1: Y Stage 2: N Stage 3: Y Stage 4: N
Ślusarkiewicz-Jarzina et al., 2005 [82]	Juvenile leaves, petioles, internodes, and axillary nodes (Callus induction, shoot induction, and rooting)	Fiber-type (5/5)	**Callus induction:** MS + dicamba (9.05 and 13.57 µM ^a^)	**Callus induction:** 52.3% (5 CV Average; petioles)	Stage 0: Y Stage 1: Y Stage 2: N Stage 3: Y Stage 4: Y
			**Shoot induction:** MS + dicamba (9.05 and 13.57 µM ^a^)	**Shoot induction:** 2.5% (cv. Silesia; petioles)	
			**Rooting:** MS + 0.57 µM IAA + 0.54 µM NAA ^a^	**Rooting:** 69.9% plantlets formed roots	
Plawuszewski et al., 2006 [67]	Axillary nodes (Direct organogenesis) Stems and roots (Indirect somatic embryogenesis)	Fiber-type (3/3)	**Callus induction:** DARIAind^+^ + NAA + BAP (concentrations N.S.)	**Callus induction:** % callusing N.S.	Stage 0: Y Stage 1: Y Stage 2: N Stage 3: Y Stage 4: N
			**Shoot proliferation:** DARIApro + NAA + BAP (concentrations N.S.)	**Shoot proliferation:** Adventitious shoot formation from axillary nodes and somatic embryo formation from stem tissue reported % N.S.	
			**Somatic embryogenesis:** DARIApro^+^ + NAA + BAP (concentrations N.S.)	**Somatic embryogenesis:** N.S.	
			**Rooting:** DARIAroot + IAA (concentrations N.S.)	**Rooting:** N.S.	
Raharjo et al., 2006 [83]	Leaves, flowers, and seedling roots, stems, and shoots (Callogenesis, callus suspension cultures)	Drug-type (0/1)	**Callogenesis:** MS + 0.56 mM mesoinositol + 29.65 µM thiamine diHCl + 4.86 pyridoxine HCl + 8.12 µM nicotinic acid + 4.52 µM 2,4-D ^a^	**Callogenesis:** Statistical analysis N.S. Callusing was greatest using flowers and seedling shoots	Stage 0: Y Stage 1: Y Stage 2: Y Stage 3: N Stage 4: N
			**Suspension culture** **(2 steps):** **Step 1:** MS (as above, aqueous; 2 weeks) **Step 2:** B5 media + 9.05 µM 2,4-D + 2.85 µM IAA + 2.69 µM NAA + 5.12 µM potassium ^a^	**Suspension culture:** Continued callus growth, no regeneration	
Wielgus et al., 2008 [68]	Cotyledons, axillary nodes, and roots (Callus induction, shoot induction, and rooting)	Fiber-type (3/3)	**Callus induction:** DARIA (ind+) + 4.65 µM kinetin + 0.27 µM NAA ^a^	**Callus induction:** Best morphogenic callus induction: stem explants (all cultivars) Statistical analysis N.S.	Stage 0: Y Stage 1: Y Stage 2: N Stage 3: Y Stage 4: N
			**Shoot induction:** DARIA (pro+) + 0.89 µM BAP + 0.16 µM NAA ^a^	**Shoot induction:** 15.56% with cotyledon explants (cv. Beniko)	
			**Rooting:** DARIA (root+) + 11.42 µM IAA ^a^	**Rooting:** Statistical analysis N.S.	
Flores-Sanchez et al., 2009 [84]	Leaves (Callus suspension cultures and somatic embryogenesis)	Drug-type (1/1)	**Suspension culture:** MS + B5 vitamins + 4.52 µM 2,4-D + 4.65 µM kinetin	**Suspension culture:** Growth rate N.S.	Stage 0: Y Stage 1: Y Stage 2: N Stage 3: N Stage 4: N
			**Somatic embryogenesis:** Media composition N.S.	**Somatic embryogenesis:** Number of embryos N.S	
Lata et al., 2010 [85]	Juvenile leaves (Callogenesis, shoot induction, and rooting)	Drug-type (1/1)	**Callogenesis:** MS + 0.5 μM NAA + 1 μM TDZ	**Callogenesis:** 93.3% response	Stage 0: Y Stage 1: Y Stage 2: N Stage 3: Y Stage 4: Y
			**Shoot induction:** MS + 0.5 μM TDZ	**Shoot induction:** 12.3 shoots/explant 96.6% response	
			**Rooting:** ½ MS + 2.5 μM IBA	**Rooting:** 10 roots/explant 96.6% response	
Farag, 2014 [28]	Juvenile leaves (Callogenesis and shoot regeneration)	Drug-type (1/1)	**Callogenesis:** B5 + 2.69 µM NAA + 22.20 µM BAP + 0.11 mM adenine hemisulfate ^a^	**Callogenesis:** 50% callusing response	Stage 0: Y Stage 1: Y Stage 2: Y Stage 3: Y Stage 4: Y
			**Shoot regeneration:** B5 + 1.44 µM GA_3_^a^	**Shoot regeneration:** 8.5 shoots/callus % regeneration N.S.	
			**Rooting:** B5 + 8.56 µM IAA ^a^	**Rooting:** 2.75 roots/explant 100% response	
Movahedi et al., 2015 [86]	Cotyledons and epicotyls (callogenesis+ shoot regeneration, rooting)	Drug-type (1/1)	**Callogenesis/shoot regeneration:** MS + 8.88 µM BAP + 2.46 µM IBA	**Callogenesis/shoot regeneration:** ~2 shoots/epicotyl % response N.S.	Stage 0: Y Stage 1: Y Stage 2: Y Stage 3: Y Stage 4: Y
			**Rooting:** MS + 0.49 μM IBA ^a^	**Rooting:** % response N.S.	
Chaohua et al., 2016 [87]	Cotyledon (callogenesis + shoot regeneration, rooting)	Fiber-type (8/8)	**Callogenesis/shoot regeneration:** MS + 1.82 μM TDZ + 1.07 μM NAA ^a^	**Callogenesis/shoot regeneration:** 3 shoots/explant (3-day-old cotyledons) 51.7% regeneration	Stage 0: Y Stage 1: Y Stage 2: N Stage 3: Y Stage 4: Y
			**Rooting:** ½ MS + 2.46–9.84 μM IBA ^a^	**Rooting:** 80% response	
Galán-Ávila et al., 2020 [88]	Hypocotyl, cotyledon and first two true leaves (direct organogenesis and rooting)	Fiber-type (5/5)	**Organogenesis:** MS + 1.82 μM TDZ + 1.07 μM NAA ^a^	**Organogenesis:** 1.49 shoots/hypocotyl 54.17% response	Stage 0: Y Stage 1: Y Stage 2: N Stage 3: Y Stage 4: Y
			**Rooting:** MS + 1.82 μM TDZ + 1.07 μM NAA ^a^	**Rooting:** ~18% rooted	
Monthony et al., 2020b [89]	Young leaves (callus induction and shoot regeneration)	Drug-type (10/10)	**Callogenesis:** MS + 0.5 μM NAA + 1 μM TDZ	**Callogenesis:** 100% response across all 10 cultivars	Stage 0: N Stage 1: N Stage 2: Y Stage 3: N Stage 4: N
			**Shoot regeneration:** MS + 0.5 μM TDZ	**Shoot regeneration:** Not achieved	

^a^ Molarity values converted from mg/L.

## Data Availability

No new data were created or analyzed in this study. Data sharing is not applicable to this article.
